# Supplementary material for: A novel channel reduction concept to enhance the classification of motor imagery tasks in brain-computer interface systems
Source: PLoS One. 2025 Oct 31;20(10):e0335511. doi: 10.1371/journal.pone.0335511 (PMC12578340; doi:10.1371/journal.pone.0335511)
Supplement: Attachment — Submitted filename: Response to Reviewers.docx [file pone.0335511.s002.docx]

**Responses of Editor and Reviewers’ comments/suggestions**

**Manuscript title: A novel channel reduction concept to enhance the classification of motor imagery tasks in brain-computer interface systems**

**Authors: Taslima Khanam¹, Siuly Siuly^1^, Kabir Ahmad^2^, and Hua Wang^1^**

**We would like to thank the editor and all reviewers for their time, constructive comments and useful suggestions which have led to a much-improved manuscript. To clearly address the amendments performed in the revised manuscript, we have quoted the specific reviewers’ comments in our responses correspondingly. In Bold are the comments from editor/reviewers, the plain blue colour text is our direct responses** **and the *Italic* is actual changes implemented, i.e. what we added, deleted, rewrote or modified. All changes to the original version of the document are now in red ink to facilitate the review process.**

**Reviewer #1: The manuscript proposed an AI-based channel selection algorithms that are both fast and accurate for identifying EEG-based motor imagery (MI) movements. A novel hybrid method combining statistical tests with Bonferroni correction is introduced to achieve an effective channel reduction rate for selecting MI task-related EEG channels and an automatic deep learning-based framework for MI task classification. The paper is well written and structured, however it needs improvement. Following are the suggestions to improve the quality of paper:**

**1. Font size and style is different at some places. It should be same in the text, paragraph, figure and tables. Follow the journal template and guidelines.**

Answer: Thank you for your valuable feedback. We sincerely apologize for the inconsistency in font size and style. We have carefully revised the manuscript to ensure uniformity in text, paragraphs, figures, and tables, adhering strictly to the journal’s template and guidelines. We appreciate your attention to detail and your efforts in improving the quality of our work.

**2. Authors have mentioned that proposed algorithm is tested and validated for two EEG datasets of5 and 7 participants, respectively. However, still the sample of overall 12 participant is too small to conclude results and claims. There are open access datasets of EEG available on the internet with adequate sample size and number of participants. The proposed algorithm should be tested and validated on larger datasets to strengthen the claims and contributions of the study.**

Answer: Thank you very much for your thoughtful comments and valuable feedback. We appreciate your concern regarding the sample size in our study. We acknowledge that a larger sample size could indeed strengthen the validity of the results and the generalizability of the findings. In response to your comment, we have now included an additional widely recognized and publicly available dataset, BCI Competition IVa Dataset 2a, which consists of data from 9 participants. This dataset is commonly used in the EEG research community and will help enhance the robustness of our experiment. We hope that this addition addresses your concern and improves the overall quality of our study.

**3. In the Table 2: 5 fold average performance results for the proposed DLRCSPNN based model, there are multiple accuracies for participants showing 100% results; this may be due to over-fitting of the classifier. This need to be verified for the results of DLRCSPRNN results as well.**

Answer: Thank you for your insightful comment regarding the potential overfitting observed in the accuracy results. To address this, we implemented several strategies to ensure the robustness of the models. First, we employed 5-fold cross-validation, ensuring that each data split was used for validation exactly once, which helps in reducing variance and improving the model's generalization. To further mitigate overfitting, we used dropout layers (0.5 rate) in both the Neural Network (NN) and Recurrent Neural Network (RNN) classifiers. These steps, combined with data standardization using StandardScaler and weight regularization with the Adam optimizer, ensured that the models would not memorize the training data but instead generalize better. We also tracked performance using standard deviation calculations, ROC curves, and AUC scores to detect any signs of overfitting. By training the models for 100 epochs and testing with various batch sizes, we further ensured that the results were not a result of overfitting. The experiments were performed on Google Colab with 2 CPU cores and 12.67 GB of RAM, without GPU acceleration, providing a consistent computational environment. We believe these measures successfully addressed overfitting and contributed to the reliable results we presented. Improvement made in the manuscript page no. 14-17.

**4. Student’s t-test and Bonferroni correction is applied to select the channels. Is there any significance statistical analysis applied on the results; accuracies?**

Answer: Thank you for your valuable feedback. Regarding the significance of the statistical analysis applied to the results, we utilized the paired t-test for comparison of the DLRCSPNN and DLRCSPRNN models’ accuracies. The results of the paired t-test revealed a T-statistic of 3.84 and a p-value of 0.001, indicating a statistically significant difference between the models. Additionally, we applied Wilcoxon signed-rank test to compare the DLRCSPNN (RC) model with the DLRCSPNN (OC) model, based on the reduced and original channel sets, respectively. This analysis provides further insight into the differences in accuracy between the two models under different conditions. We hope this clarifies the statistical methods used for the analysis of the results and accuracies. Pge no. 22-23, 26

**5. In Table 4. Architectural comparison of the different models are discussed, however the hardware details are not described on which both the algorithms are computed.**

Answer: Thank you for your insightful comment. Regarding the hardware details on which both algorithms were computed, we used Google Colab, a cloud-based platform, with the following specifications: 2 CPU cores, 12.67 GB of RAM, and no GPU acceleration. The computational environment is virtualized, and the experiments were conducted without dedicated GPU support, which may affect processing time for resource-intensive tasks. We hope this additional context clarifies the hardware setup used for the computations of both algorithms. Page no.16.

**Reviewer #2: This paper, in its current form, lacks the essential elements of a well-structured and scientifically rigorous study, making it difficult to consider it a strong contribution to the field. Significant issues across multiple sections need to be addressed:**

**1. Introduction and Literature Review: The introduction section is missing a detailed literature review, particularly for the classification methods discussed. A comprehensive background and context are essential to highlight the novelty** **and relevance of the study.**

Answer: We greatly appreciate your insightful feedback. In response to your comment, we have addressed the need for a more comprehensive literature review in the introduction, with a particular focus on the classification methods used in the study. The expanded literature review is now detailed on pages 2-4 of the manuscript.

In this section, we have provided an in-depth discussion of key motor imagery task classification methods used in EEG analysis. We specifically explored various channel selection techniques, including genetic algorithms, common spatial patterns, and other methods, outlining both their strengths and limitations. By reviewing these existing approaches, we aim to demonstrate the novelty and relevance of our study, particularly in relation to our AI-based channel selection method, which combines statistical tests with Bonferroni correction to improve classification accuracy while reducing computational complexity.

We believe that this more detailed background helps to clearly position our work within the broader context of BCI technology, and we hope this revision effectively highlights the significance of our contributions.

**2. Figure Quality:**

**o Figure 1: The image is blurry and appears AI-generated. Its quality needs significant improvement to enhance clarity and readability.**

**o Figures 2 and 3: These figures are also blurry and lack detailed**

Answer: We appreciate your observation regarding Figure 1, 2 and 3. We have now improved its quality to ensure better clarity and readability. Page 5, 7, 12.

**3. Dataset Details: o Clarify the distinction between Dataset 1 (imaginary motor tasks involving the right hand and foot) and Dataset 2 (motor acquisition).**

**o Provide justification for why two participants performed left-hand and foot activities, while others focused on the right-hand tasks.**

**o Discuss the implications of such variations in task allocation on the results.**

Answer: Thank you for your valuable feedback. We appreciate your comments and are happy to provide clarification regarding the datasets and task allocation. In the manuscript, page no. 5 to 6.

BCI Competition III Dataset IVa:

This dataset is focused on motor imagery tasks involving the right hand and right foot. Participants were asked to imagine movements of either their right hand (class 1) or right foot (class 2), with no actual physical movement involved. EEG data were recorded using 118 electrodes following the 10/20 international system. The dataset includes data from five participants, each completing 280 trials, with each trial lasting 3.5 seconds. The primary aim of this dataset is to study the brain's response to imagined motor tasks, which can be used in applications such as brain-computer interfaces (BCI). Importantly, no physical movements were performed by the participants, as the focus was solely on motor imagery.

BCI Competition IV Dataset 1:

Unlike Dataset IVa, BCI Competition IV Dataset 1 involves both motor imagery and motor execution tasks. Participants performed tasks involving their left hand, right hand, and foot. EEG data were recorded using 59 channels from seven participants. During the trials, participants either imagined (motor imagery) or physically executed (motor execution) the movements of different body parts. The goal of this dataset is to investigate brain activity during both motor imagery and actual movement, focusing on the differences between these two types of brain activity. This dataset is particularly relevant for real-world applications, such as controlling prosthetics and other BCI systems that require accurate motor command decoding.

Regarding your question about why certain participants performed left-hand and foot activities, while others focused on right-hand tasks, the dataset does not provide explicit reasons for these assignments. From the available documentation, it appears that the task allocation was based on the participants’ choices or experimental design, but further details are not provided by the dataset creators.

BCI Competition IV Dataset 2a:

This dataset involves motor imagery tasks across a broader range of body parts, including the left and right hand, both feet, and the tongue. The dataset includes data from nine participants, with recordings made during two sessions. Each session contains 288 trials, divided into six runs. The first session contains labeled data for model training, while the second session contains unlabeled data for evaluation. EEG signals were recorded using 22 Ag/AgCl electrodes and three EOG channels, sampled at 250 Hz. The focus of this dataset is on analyzing motor imagery for different body parts, making it valuable for developing BCIs that can decode complex motor imagery tasks across a variety of body parts.

Distinctions:

1. BCI Competition III Dataset IVa focuses only on motor imagery tasks involving the right hand and right foot, whereas BCI Competition IV Dataset 1 includes both motor imagery and motor execution tasks involving the left hand, right hand, and foot.
2. BCI Competition IV Dataset 1 uses 59 channels for data collection, while BCI Competition III Dataset IVa uses 118 channels, making Dataset IVa higher in channel count.
3. BCI Competition IV Dataset 2a includes motor imagery tasks for a wider range of body parts (left and right hand, both feet, and tongue) and has a larger number of participants (nine participants), compared to Dataset 1, which uses only seven participants.

In summary, the datasets differ in the type of tasks (motor imagery vs. motor execution), the number of body parts involved, the number of participants, and the number of EEG channels used. We hope this clears up the distinctions between the datasets and the rationale for task allocation.

**4. Methodological Concerns:**

**o Line 151: The sentence mentioning "might lose important information in discarded channels" raises concerns. Results should be compared with all channels to confirm whether the channel selection method inadvertently excludes useful information.**

Answer: We appreciate your valuable feedback and the opportunity to address your concern regarding the potential loss of important information when discarding channels. In response, we performed a direct comparison between the full channel set and the selected channel set for the DLRCSPNN model, specifically evaluating both accuracy and computational time in the manuscript page no.24-26.

*Additionally, we conducted experiments using the original channels for all subjects and compared the results with the DLRCSPNN algorithm across three datasets. As shown in Figures 12 and 13, DLRCSPNN (RC) consistently outperformed DLRCSPNN (OC) in terms of both time efficiency and accuracy. For subjects like 'a', 'b', and 'c', DLRCSPNN (RC) demonstrated significantly faster processing times and higher accuracy. For example, subject 'a' showed 98% accuracy for RC versus 97% for OC, and subject 'b' showed 99% accuracy for RC versus 98% for OC. Even in subjects like 'f', 'g', and 'aa', RC maintained a slight accuracy advantage, such as 97% versus 96% in subject 'g'. In more challenging cases, such as 'aw', 'ay', and A-series subjects, RC continued to be faster while maintaining a marginal edge in accuracy (e.g., 60 seconds for RC versus 65 seconds for OC in 'aw').*

*These findings suggest that DLRCSPNN (RC) offers a more efficient and reliable model for most subjects, providing a well-balanced trade-off between speed and performance. Therefore, DLRCSPNN (RC) is the preferred choice over DLRCSPNN (OC). To statistically validate this difference, we performed a Wilcoxon signed-rank test comparing the two models. The test resulted in a statistic of 50.5 and a p-value of 0.0419, which is below the conventional significance threshold of 0.05. This indicates a statistically significant difference between the full channel set of model and the reduced channel set of model, suggesting that reducing the number of channels notably impacts the accuracy.*

We believe this additional analysis provides a clear justification for the channel selection method used and assures that the results are reliable and well-supported. Thank you for bringing this to our attention.

**o Line 198: Correct the typo where "=1=1" is mentioned.**

Answer: We appreciate your attention to detail. The typo "=1=1" in Line 198 has been corrected. Thank you for bringing this to our attention. Page no. 10.

**o Details on the DLRCSP algorithm are missing. Explain how the algorithm is designed and applied in the study.**

Answer: Thank you for your insightful comment. We have now provided a detailed explanation of the DLRCSP algorithm in the manuscript to address your concern in page no. 9-10.

**5. Neural Network Representation:**

**o Figure 3: The representation is unclear. It suggests input data is fed to all three dense layers separately, which is logically inconsistent. Recheck the flow of information within the network layers and clarify the purpose of lines joining the blocks.**

Answer: We appreciate your attention to detail. Figure 3 has been corrected.

**6. Experimental Setup and Results:**

**o Line 238: Merely training the model on k−1k-1k−1 subsets (with only 42 samples) is insufficient. Justify how the training data is adequate for robust model performance.**

Answer: Thank you for your constructive comment. We apologize for the oversight regarding the use of only k−1 subsets (with 42 samples) for training the model. After carefully reviewing the methodology, we have made the necessary corrections. We have adjusted the dataset and the training procedure to ensure that the model is trained on an adequate amount of data for robust performance. We appreciate your understanding and have updated the manuscript to reflect these improvements.

**o When comparing the proposed model with RNN algorithms, ensure the number of neurons and sequence of layers are consistent. Differences in network architecture can significantly impact results and invalidate comparisons.**

Answer: Thank you for your thoughtful feedback. We have carefully considered your comment regarding ensuring consistency in the network architecture when comparing the proposed model with RNN algorithms. To address this, we have ensured that the number of neurons and sequence of layers are consistent across both the DLRCSPNN and RNN models page no. 10-12 in manuscript.

*In this study, we have developed an NN model to perform classification on the generated dataset. The proposed NN model consists of multiple dense layers with dropout for regularization. Specifically, it includes three dense layers: the first two with 64 neurons each, followed by dropout layers with a 50% rate to prevent overfitting. The third dense layer has 32 neurons, and all dense layers use the ReLU activation function to introduce non-linearity. The final classification layer consists of two output units corresponding to the binary classification task and uses the softmax activation function to produce probability scores.*

*Recurrent Neural Network RNN: Recurrent Neural Networks (RNNs) are a type of neural network designed to process sequential data, such as time series or natural language. They were introduced by David Rumelhart and Geoffrey Hinton in 1986. For the RNN model in this study, we implemented an architecture with 64 LSTM units in the first layer, followed by a dense layer with 64 neurons, and additional dropout layers (with a 50% rate) to prevent overfitting. The LSTM layer is responsible for capturing long-range dependencies in the sequential data, which is essential for EEG signal processing. The architecture also includes another dense layer with 32 neurons, using ReLU activation, followed by a final output layer with 2 neurons (for binary classification), using softmax activation to produce probability scores. The model is trained using the sparse categorical cross-entropy loss function and the Adam optimizer, which adapts the learning rate during training. This architecture ensures that the number of neurons and the sequence of layers align with the neural network (NN) model, facilitating a fair and meaningful comparison. LSTM-based RNNs are chosen due to their ability to effectively handle long-range dependencies in sequential data, which makes them particularly well-suited for EEG classification tasks. While GRUs could offer faster computation, LSTMs were selected for their proven effectiveness in EEG signal processing. The decision to focus on LSTM models provides a consistent evaluation framework for this study, with future research exploring comparisons with GRUs or other architectures to further validate the approach*

*This adjustment ensures that both models have a similar architecture, with consistent numbers of neurons and layer sequences, enabling a fair and meaningful comparison. Additionally, both models were evaluated using 5-fold cross-validation to assess accuracy, computational time, and robustness, ensuring that the comparison is valid and not affected by architectural discrepancies.*

We hope this clarifies how we have aligned the architectures and addressed the concern about potential inconsistencies.

**o Results are only compared with a single RNN algorithm (LSTM). Provide a rationale for choosing RNN and LSTM and explain why comparisons with other architectures (e.g., GRU) are omitted.**

Answer: Thank you for your valuable comment. In this study, we chose to compare the proposed model with a single RNN architecture, specifically LSTM (Long Short-Term Memory), as it is well-suited for handling sequential data like EEG signals, known for capturing long-term dependencies (W.Yuan et al, 2024). LSTM has been extensively used in EEG classification tasks, particularly for motor imagery, due to its superior ability to manage temporal dependencies (Li et al., 2020).

While GRUs are computationally more efficient, prior research suggests that LSTMs perform better in complex EEG classification tasks due to their ability to retain information over longer sequences (Cho et al., 2014). While GRUs are computationally more efficient, prior research suggests that LSTMs perform better in complex EEG classification tasks due to their ability to retain information over longer sequences

**7. Subject-Specific Analysis:**

**o Figure 7: Results for subject "ay" are missing when using RNN. Ensure all subjects are included in the analysis for consistency.**

Answer: Thank you for pointing this out. We apologize for the oversight regarding the missing results for subject "ay" in Figure 7 when using the RNN mode. We have corrected this issue in the manuscript (page no. 20), and all subjects, including "ay," are now included in the analysis for consistency. Thank you for your understanding.

**o Comparisons between datasets (motor imagery vs. motor acquisition) are problematic as the datasets inherently differ in nature, which could skew results.**

Answer: Thank you for your insightful comment. *We acknowledge that motor imagery (MI) and motor execution (ME) tasks inherently differ in nature, and direct comparisons between these datasets may introduce confounding factors. However, dataset comparisons involving MI tasks across different EEG datasets are a well-established practice in BCI research. Specifically, Qin et al. (2025) introduced a cross-domain-based channel selection method for MI classification, demonstrating that MI datasets from different sources can be analyzed together under a standardized preprocessing and feature extraction framework. Their study successfully applied cross-domain learning techniques to datasets with different subjects and recording conditions, validating that MI datasets can be benchmarked against each other when methodological consistency is maintained.*

*In alignment with this* prior work, we ensured that our dataset comparisons followed a task-specific approach, where MI tasks were analyzed separately across datasets. Additionally, our preprocessing *steps—including artifact removal, frequency filtering, feature extraction, and normalization—were applied uniformly across datasets to maintain comparability. As also demonstrated by Qin et al. (2025), domain adaptation techniques can further reduce dataset discrepancies, and we propose incorporating such methods in future studies to enhance generalizability. Given this context, we believe that our dataset comparisons are methodologically sound and consistent with established BCI research practices.*

**8. Comparison and Benchmarking:**

**o Comparative results are not adequately presented in tables or bar charts. Include comparisons with benchmark algorithms or methodologies to demonstrate the performance of the proposed approach.**

**o Ensure all bar charts include comparative results for clarity.**

Answer: Thank you for your valuable suggestions. We have now included comprehensive comparative results in bar charts to clearly demonstrate the performance of our proposed approach against benchmark algorithms. The bar charts have been updated to ensure all necessary comparisons are presented for better clarity. We appreciate your feedback and believe these additions enhance the readability and impact of our findings.

**9. Discussion Section:**

**o The discussion section does not adequately support the results or compare them with findings from previous studies. Expand this section to include critical analysis and comparisons with related works.**

Answer: Thank you for your valuable feedback. In response to your comment, we have expanded the discussion section to include a more detailed critical analysis and comparison of our results with previous studies in the manuscript in page no. 26-28.

**10. Methodology and Results Flow:**

**• Experimental setups mentioned in the results section should be moved to the methodology section for better structure and flow.**

Answer: Thank you for your helpful suggestion. In response to your comment, we have moved the experimental setups from the results section to the methodology section to improve the structure and flow of the manuscript in page no. 13-14. We believe this revision enhances the clarity and organization of the paper.

**References:**

Yuan W, Fu D, Hu W. The Research Focuses on the Classification and Recognition of Speech Imagery EEG Signals Based on a Fusion Network Autoencoder. In2024 5th International Conference on Artificial Intelligence and Computer Engineering (ICAICE) 2024 Nov 8 (pp. 223-227). IEEE.

Hochreiter, S., & Schmidhuber, J. (1997). Long short-term memory. *Neural Computation, 9*(8), 1735–1780.

Li, Y., Li, H., & Wang, S. (2020). A review of deep learning methods for EEG classification. *Computers in Biology and Medicine, 122*, 103845.

Cho, K., Van Merriënboer, B., Gulcehre, C., Bahdanau, D., Bougares, F., Schwenk, H., & Bengio, Y. (2014). Learning Phrase Representations using RNN Encoder–Decoder for Statistical Machine Translation. *EMNLP 2014*.

Qin Y, Zhang L, Yu B. A cross-domain-based channel selection method for motor imagery. Medical & Biological Engineering & Computing. 2025:1-11.
