# Supplementary material for: A novel channel reduction concept to enhance the classification of motor imagery tasks in brain-computer interface systems
Source: PLoS One. 2025 Oct 31;20(10):e0335511. doi: 10.1371/journal.pone.0335511 (PMC12578340; doi:10.1371/journal.pone.0335511)
Supplement: Attachment — Submitted filename: Response to reviewer.docx [file pone.0335511.s003.docx]

**Responses of Editor and Reviewers’ comments/suggestions**

**Manuscript title: A novel channel reduction concept to enhance the classification of motor imagery tasks in brain-computer interface systems**

**Authors: Taslima Khanam¹, Siuly Siuly^1^, Kabir Ahmad^2^, and Hua Wang^1^**

**We would like to thank the editor and all reviewers for their time, constructive comments and useful suggestions which have led to a much-improved manuscript. To clearly address the amendments performed in the revised manuscript, we have quoted the specific reviewers’ comments in our responses correspondingly. In Bold are the comments from editor/reviewers, the plain blue colour text is our direct responses and the *Italic* is actual changes implemented, i.e. what we added, deleted, rewrote or modified. All changes to the original version of the document are now in red ink to facilitate the review process.**

**Response to Reviewer #1:**

**We thank the reviewer for acknowledging the improvements made and for providing additional constructive feedback. We have carefully addressed each of the remaining concerns as follows:**

**Reviewer #1: The authors have improved the manuscript significantly, however following concerns still need to be addressed.
1. Font Size and style: This still need to addressed, it is recommended to review the complete thoroughly and use same font style in text, tables and figures. It is evident that font style of references is different from other text in the manuscript. Table 5 has multiple font styles.**

Response: We sincerely apologize for the oversight regarding inconsistent font usage. The manuscript has now been thoroughly reviewed to ensure uniform font style (e.g., Times New Roman) and size (12 pt for main text, appropriately scaled in tables and figures) throughout the document. Special attention was given to the following:

- All tables, including Table 5, have been reformatted to ensure consistency in font style and size.
- The reference list has also been updated to match the main body text in both font and formatting.

We believe this correction improves the overall professionalism and readability of the manuscript.
**2. Figure size and aspect ratio: the figure font style and size should be same as text, also keep aspect ratio of figure readable. Moreover, figure 1 text (flowchart) is not readable.**

Response: All figures have been revised to ensure consistent font style and size, in alignment with the manuscript text. The aspect ratios have also been adjusted to enhance clarity and readability. Specifically:

- Figure text (axis labels, legends, annotations) now uses a font size and style that matches the manuscript.
- Figure 1 (flowchart) has been recreated with a clearer layout, larger font, and optimized resolution for easy readability.

All figures have been checked for consistent aspect ratio and alignment across the document.
**3. Results of Wilcoxon signed-rank test should be included in the manuscript as separate heading “statistical significance”**

**Response:** As recommended, we have added a subsection titled “Statistical Significance” under the Results section. This subsection reports the results of the Wilcoxon signed-rank test comparing the performance of DLRCSPNN models using retained channels (RC) versus original channels (OC). The added text is as follows:

***Statistical Significance***

*To statistically validate this difference, we performed a Wilcoxon signed-rank test comparing the two models. The test resulted in a statistic of 50.5 and a p-value of 0.0419, which is below the conventional significance threshold of 0.05. This indicates a statistically significant difference between the full channel set and the retained channel set, suggesting that reducing the number of channels notably impacts the accuracy. Thus, the choice of channels is crucial for the model's performance, and channel reduction has a measurable effect on accuracy.*

**Reviewer #2: Channel Selection:

The proposed channel reduction method is novel but lacks comparison with existing channel selection techniques.
The paper does not specify which or how many channels were selected from each dataset.
Suggestion: Include comparative results with standard methods and clearly state selected channels per dataset.**
**Response:** We thank the reviewer for their valuable comment regarding the evaluation of the proposed channel selection approach.

In response, we have revised the manuscript to clearly specify the number of channels selected per subject across all three datasets. As presented in Table 2 (DLRCSPNN model) and Table 3 (DLRCSPRNN model), we report the Original Channels (OC), the Retained Channels (RC), and the Channel Reduction Ratio (CRR) for each subject. For instance, subject *aw* had 118 original channels, from which only 27 were retained (CRR = 0.22), while subject *A06* had 22 original channels and only 5 were retained (CRR = 0.22). These values are now explicitly included in the results section to ensure transparency and reproducibility.

To address the request for comparative evaluation, we have included three comparative bar charts (Figures 14–16) that benchmark the proposed method against a range of existing channel selection-based approaches across the same datasets. These figures present the classification accuracy as bar heights, while the numeric values on top of each bar indicate the number of channels retained by each method. The proposed method consistently achieves competitive or superior accuracy while retaining fewer EEG channels compared to prior works. For example, in Figure 14 (Dataset 1), the proposed method retained only 5–12 channels, achieving accuracy between 90.03% and 94.22%, while other methods such as Tang et al. retained up to 22 channels for similar or lower accuracy. A similar trend is observed in Figures 15 and 16 across subjects in Datasets 2 and 3.

In addition, the comparative performance of DLRCSPNN and DLRCSPRNN further validates the stability and effectiveness of our selection strategy. The DLRCSPNN model yielded higher average accuracy and lower variability across different batch sizes (32, 64, 128), with the highest performance typically observed at batch size 32. This suggests that the proposed channel selection method supports both robustness and computational efficiency in training, especially under resource-constrained conditions.

These results collectively demonstrate that our channel reduction method achieves strong performance with significantly fewer channels, offering both practical and computational advantages. All channel selection results per subject are now detailed in the manuscript and the corresponding figures and tables have been updated accordingly.
**Dataset Details:
It’s unclear if all three datasets are binary classification problems.
Suggestion: Clarify class details for each dataset.**
**Response**

Thank you for your valuable suggestion. We have clarified all datasets were structured for binary classification tasks, either initially or through a subset selection, ensuring consistency across all datasets for the purposes of our analysis. The class details for each dataset used in the study below:

*In our study, we utilized three publicly available datasets, dataset 1: BCI Competition III Dataset IVa, dataset 2: BCI Competition IV- dataset 1 [32, 33] and dataset 3: BCI competition IV dataset 2a. All datasets are accessible via the following links,* [*Data Set IVa for the BCI Competition III (bbci.de)*](https://www.bbci.de/competition/iii/desc_IVa.html)*,* [*Data Set 1 for the BCI Competition IV (bbci.de)*](https://www.bbci.de/competition/iv/desc_1.html) *and* [*BCI Competition IV (bbci.de)*](https://www.bbci.de/competition/iv/)*. Access to these datasets is granted upon agreeing to the terms and conditions outlined in the “Download of data sets” section. We followed the same procedure to obtain access for this study. This study involves secondary data analysis of publicly available, de-identified data. In the original data collection, all participants provided informed, written consent for the use of their data in research under the assurance of confidentiality. Additionally, the datasets were fully anonymized by the original data providers before being made accessible, ensuring no identifiable information was included. As a secondary analysis, no participants in our study are directly connected to our research team, and no additional consent was required for our specific use. Therefore, this research does not require additional ethical approval. Dataset 1 wasgathered from five healthy, labeled subjects (aa, al, av, aw, and ay) who performed motor imagery (MI) tasks involving their right hands (class 1) and right feet (class 2) forming a* ***binary classification*** *problem. The subjects were seated in a comfortable chair with armrests during the trials. The dataset includes EEG-based MI data from the first four sessions without feedback, recorded using 118 electrodes according to the 10/20 international system. Each subject participated in 140 out of a total of 280 trials, performing one of the two MI tasks (right hand or right foot) for 3.5 seconds per trial. The training set consisted of 168, 224, 84, 56, and 28 trials for subjects aa, al, av, aw, and ay, respectively, with the remaining trials forming the test set.*

*Dataset 2, sourced from BCI Competition IV, also represents a* ***binary classification setup.*** *It includes EEG data from59 channels across 7 participants. Two participants performed left hand (L) and feet (F) MI tasks, while the other participants engaged in right hand (R) and left hand (L) activities. During the first two runs, visual cues corresponding to left, right, or down arrow keys were presented on the screen for 4 seconds, guiding the subjects to execute the appropriate MI tasks. These visual cues were followed by a fixed cross and 2 seconds of a blank screen. The recordings were captured using an Ag/AgCl electrode cap, with a sampling rate of 100 Hz, and the calibration data consisted of two runs, each containing 100 single observations*

*Dataset 3, BCI Competition IV dataset 2a, involves motor imagery tasks (left/right hand, both feet, and tongue) categorized into four classes. The dataset includes EEG data from 9 participants across two sessions, with each session consisting of 288 trials divided into six runs. One session provides labeled data for model training, while the other offers unlabeled data for evaluation. Participants were seated comfortably, with trials beginning with a fixation cross and an auditory warning. EEG signals were recorded using 22 Ag/AgCl electrodes and three EOG channels, sampled at 250 Hz, with filters applied to remove noise. Signal analysis focuses on motor imagery sequences. For analytical purposes, in this study,* ***we extracted a binary subset*** *by selecting only right hand (Class 1) and feet (Class 2) trials to maintain consistency with the other datasets and align with our binary classification framework.. Therefore, for all analyses presented in this study, each dataset was structured and processed for* ***binary classification*** *tasks. All datasets are publicly available online, and informed consent for the publication of data was obtained from each participant at the time of collection. As no personal identification information was included, no ethical approval was required for our study. Table I summarizes the demographic data of participants across the datasets, and Fig. 2 illustrates an example pattern of EEG signals from channel C3 of both datasets after applying Butterworth filter and our developed channel selection method. The signals exhibit characteristic fluctuations in amplitude over time, with values constrained within the -50 to 50 range, for dataset 1 and -20 to 20 range for dataset 2, indicating effective filtering*.

**Figure 10 Clarity:
The combined plot is unclear.
Suggestion: Plot DLRCSPNN and DLRCSPRNN results separately for better clarity.**

Response: Thank you for your valuable suggestion. We acknowledge that the combined plot may have caused some clarity issues. To address this, we have now separated the DLRCSPNN and DLRCSPRNN results into individual plots. This change ensures that each method’s performance is clearly presented and easily distinguishable, allowing for a more focused comparison. We have updated Figure 10 accordingly and resubmitted the revised version for your review.
**Efficiency Claims (Line 550):
Efficiency isn’t quantified in results.
Suggestion: Add computational time comparison with baseline NN and RNN (without DLRCSP). Expand Figure 11 accordingly.**
Response:

Thank you for your suggestion. While baseline NN and RNN models without DLRCSP were not included in our analysis—as they do not incorporate any channel selection and thus are not directly comparable in structure or objective—we have quantified efficiency by comparing DLRCSPNN with DLRCSPRNN in terms of both execution time and accuracy (see revised Figure 11). Additionally, we compared DLRCSPNN using reduced channels (RC) versus original channels (OC) across all subjects (Figures 12 and 13), demonstrating lower execution time and higher accuracy with Retained Channels. These results substantiate our claims of improved computational performance.

**Unsupported Claim (Line 642):
No comparative accuracy results are shown for existing channel selection methods.
Suggestion: Remove or support the claim with data.
Discussion – Lower Accuracy (Figures 12–16):
Other studies show higher ACC and RC.
Suggestion: Discuss possible reasons for lower performance of the proposed method.**

**Response:**

Thank you for pointing this out. We have revised the manuscript to fully support the claim with comparative accuracy results against existing channel selection methods. Specifically, in the manuscript Figures 14–16, which clearly display the classification accuracy (as bar heights) and the number of retained EEG channels or selected channel (as numeric labels) for each subject across three benchmark datasets.

- In Figure 14 (Dataset 1), DLRCSPNN achieves higher accuracy with fewer channels compared to all baseline methods. For example, subject aa achieves 99.84% accuracy with 49 channels, outperforming *Tiwari & Chaturvedi (89.34%)* and *Gaur et al (75.89%)* with 49 and 31 channels respectively.
- In Figure 15 (Dataset 2), subject a achieves 99.33% accuracy using 37 channels, while competing methods like *Du et al* and *Tang et al* achieve lower accuracy despite using more channels.
- In Figure 16 (Dataset 3), DLRCSPNN achieves 92.08% with only 6 channels for subject A02, significantly surpassing *Khalid et al's* method which achieves 73.41% with the same number of channels.

These visual and numerical comparisons across datasets directly validate and support the original claim. The corresponding discussion section (Section 4) and figure captions have also been updated to reflect this.

We appreciate the reviewer’s observation. In cases where the proposed DLRCSPNN method shows slightly lower classification accuracy compared to certain existing methods (e.g., A03 or A08 in Dataset 3), this is primarily due to our aggressive channel pruning strategy, which aims to reduce the number of EEG channels to a minimal yet effective subset. In the manuscript, it is mentioned like below:

*In a few instances, such as subjects A03 or A08, our method exhibited slightly lower accuracy compared to specific high-performing baselines. We attribute this to our model’s aggressive pruning, which, while highly effective for reducing computational overhead, may occasionally sacrifice marginal accuracy in favor of generalizability and resource efficiency. Additionally, EEG signals are inherently subject-specific and nonstationary, which can influence per-subject performance under a reduced-channel setting.*

These justifications have now been integrated into the revised Discussion section, alongside references to Figures 12–16 and Table 2.
