# Supplementary material for: A novel channel reduction concept to enhance the classification of motor imagery tasks in brain-computer interface systems
Source: PLoS One. 2025 Oct 31;20(10):e0335511. doi: 10.1371/journal.pone.0335511 (PMC12578340; doi:10.1371/journal.pone.0335511)
Supplement: Attachment — Submitted filename: Response to reviewer_2nd minor rivision.docx [file pone.0335511.s004.docx]

**Responses of Editor and Reviewers’ comments/suggestions**

**Manuscript title: A novel channel reduction concept to enhance the classification of motor imagery tasks in brain-computer interface systems**

**Authors: Taslima Khanam¹, Siuly Siuly^1^, Kabir Ahmad^2^, and Hua Wang^1^**

**We would like to thank the editor and all reviewers for their time, constructive comments and useful suggestions which have led to a much-improved manuscript. To clearly address the amendments performed in the revised manuscript, we have quoted the specific reviewers’ comments in our responses correspondingly. In Bold are the comments from editor/reviewers, the plain blue colour text is our direct responses. All changes to the original version of the document are in track change mode to facilitate the review process.**

**Response to Reviewer**

**We thank the reviewer for acknowledging the improvements made and for providing additional constructive feedback. We have carefully addressed each of the remaining concerns as follows:**
**Reviewer #1: Following are the comments to improve the manuscript**

**1. Again the Figure-1 needs improvement. The fonts in the figure are not readable and images are blur. If the idea is to present the methodology/approach, then simple block diagram may work better here, however every step should include the text representation of methods.**

Response: We sincerely apologize for the oversight regarding Figure-1. In line with your valuable suggestion, we have redrawn Figure-1 as a clear, high-resolution block diagram. Each step now explicitly includes a text label describing the corresponding method. This ensures readability, consistency, and a clearer presentation of the methodology/approach.

**2. Same as highlighted previous, the aspect ratio and size of the all the figures are different. Make sure to use same fonts, size and style in all figures.**

Response: Thank you for highlighting this issue. We have carefully reformatted all figures to ensure consistent aspect ratios, font types, sizes, and styles throughout the manuscript. This has improved both readability and the professional appearance of the figures. However, Figure 3 could not be adjusted to the exact same size as the others due to its overview structure.

**3. Thoroughly review and correct the references as per author’s guidelines. For example reference number 44-47 are incomplete.**

Response: We appreciate your observation regarding the reference list. We have thoroughly reviewed and corrected the references according to the journal’s author guidelines. In particular, references 44–47 have been completed and formatted properly. We also re-checked the entire reference list to ensure consistency and completeness.
